# STK33 overexpression in hypopharyngeal squamous cell carcinoma: possible role in tumorigenesis

**DOI:** 10.1186/s12885-015-1009-3

**Published:** 2015-01-21

**Authors:** Lingyan Huang, Chen Chen, Guodong Zhang, Yuanrong Ju, Jianzhong Zhang, Haibo Wang, Jianfeng Li

**Affiliations:** 1 Department of Otolaryngology-Head and Neck Surgery, Provincial Hospital Affiliated to Shandong University, Jingwu Street 324, Jinan, 250021 P.R. China; 2 Department of Pathology, General Hospital of Ningxia Medical University, Yinchuan, 750004 P.R. China; 3 Intensive Care Unit, Provincial Hospital Affiliated to Shandong University, Jinan, 250021 P.R. China; 4 Department of Pathology, Medical College, Shandong University, Jinan, 250021 P.R. China

**Keywords:** Serine/threonine kinase 33, Fadu cells, ERK1/2, Tumorigenesis

## Abstract

**Background:**

The role of serine/threonine kinase 33 (STK33) gene in tumorigenesis is still controversial. This study was aimed to investigate whether STK33 had the effect on hypopharyngeal squamous cell carcinoma (HSCC) and relevant genes, as well as the potential relation to ERK1/2 pathway.

**Methods:**

Immunohistochemistry was performed to investigate STK33 expression in human HSCC specimens. MTT, immunofluorescence, clone formation and matrigel invasion assays were employed to detect the effects of STK33 knockdown (STK33-RNAi) and/or PD98059 on major oncogenic properties of a HSCC cell line (Fadu), while, real-time PCR and western blot were used to examine the expressions of relevant genes.

**Results:**

STK33 was over-expressed in HSCC specimens, which was significantly associated with certain clinicopathological parameters. STK33-RNAi in Fadu cells resulted in inhibition of proliferation, induction of apoptosis, reduction of clone formation, and decline in the migration and invasion. These effects were potentiated by administration of PD98059. Mechanistic studies revealed that STK33-RNAi led to an increase in Caspse-3, Nm-23-H1 and E-Cadherin expressions and a reduction in Bcl-2, Ki-67 and Vimentin expressions. Moreover, PD98059 significantly reduced both ERK1/2 and STK33 expressions in Fadu cells.

**Conclusions:**

STK33 is a potential oncogene and a promising diagnostic marker for HSCC. STK33 may promote tumorigenesis and progression of HSCC, and serve as a valuable molecular target for treatment of HSCC.

## Background

Hypopharyngeal squamous cell carcinoma (HSCC), which originates from a subsite of the upper aerodigestive tract, accounts for 2-6% of head and neck cancers, the 6th most common cancer in the world [1,2]. As this type of cancer is initially diagnosed at an advanced stage, thus, therapy for this tumor is one of the most formidable challenges in human malignancies. As such, studies of the molecular biology of this disorder are key issues to get insight into the mechanistic underpinnings of how incipient cells become tumorigenic and malignant ultimately, which may, in turn, lead to offer novel therapeutics to patients with advanced hypopharyngeal cancer, with more expectation of both survival improvement and maximum preservation of hypopharyngeal function. While numerous genetic and epigenetic factors have been found to be involved in the pathogenesis of HSCC, the molecules that participate in tumorigenesis of this kind of carcinoma are still known little. More recently, compelling evidence has indicated the involvement of serine/threonine kinase 33 (STK33) gene in tumorigenesis [3,4].

STK33 gene encodes a serine/threonine kinase [5], which is subsequently identified as a member of the calcium⁄ calmodulin dependent kinase (CAMK) family [6]. Of note, STK33 involves in the “synthetic lethal” process of a variety of tumor cells, which depends on the Ras oncogene [4]. This finding implies that STK33 might possess an important value in molecular target therapy for KRAS-dependent tumors. However, a new study demonstrates that STK33 activity is nonessential in KRAS-dependent cancer cells [7,8]. The pro and con evidence indicates that the role of STK33 in tumor cells remains controversial and, from another angle, reflects that the mechanism underlying the action of STK33 to alter the nature of tumor may be much more complex [7-9].

In comparison to the other members of CAMK family, which are well known to phosphorylate a wide range of substrates and regulate numerous cellular functions [10], the precise mechanism underlying the actions of STK33, especially, on survival or signaling pathways on tumor cells, however, has been studied sparingly as yet. It has been confirmed that mitogen-activated protein kinase (MAPK) system, which comprises a cluster of a serine/threonine protein kinases, represents one of the most classical signal transduction pathways [11]. Almost all eukaryotic cells possess multiple MAPK pathways, which generally regulate a variety of cell activities in response to different stimuli. Particularly, extracellular signal-regulated protein kinases 1 and 2 (ERK1/2), the members of the MAPK family, is able to mediate cell proliferation, survival, differentiation and motility etc. [12], and thus, is widely used throughout evolution in many physiological and pathological processes.

To date, no study has been conducted to investigate STK33 expression in human HSCC as well as the relationship between the STK33 gene and ERK1/2 pathway in carcinogenesis of HSCC. With these points in mind, we firstly examined the expression of STK33 in human HSCC specimen and its association with clinicopathological characteristics and, then, explored whether STK33 gene could affect the biological traits, such as apoptosis, proliferation, metastasis and invasion, as well as epithelial mesenchymal transition (EMT) in Fadu cells, a cell line of human HSCC, and its relationship with ERK1/2 signaling pathway.

## Methods

### Carcinoma specimens

HSCC specimens and corresponding adjacent normal tissues were obtained from 30 patients who underwent operations at General Hospital of Ningxia Medical University (Yinchuan, P.R. China) from 2010 to 2013. Patients had received neither adjuvant chemotherapy nor radiotherapy prior to surgery. The pathological analysis was conducted according to criteria issued by World Health Organization (WHO) [13]. Tumor clinical stage was defined according to criteria by American Joint Committee on Cancer (AJCC) [14]. All the detailed information and clinicopathological parameters of patients in this series were provided in Table 1. This study was reviewed and approved by the Institute Research Ethics Committee of General Hospital of Ningxia Medical University, Yinchuan, P. R. China. All patients provided written informed consent for this research.

**Table 1:** **HSCC patients’ clinicopathological characteristics and IHC staining for STK33**

	N	STK33 IHC score^a^	*P*value^b^
**Age**			
<60 years	13	13.69 ± 4.23	0.8988
≥60 years	17	14.12 ± 2.98	
**Sex**			
Male	28	13.96 ± 3.65	0.8255
Female	2	13.50 ± 2.12	
**Differentiation** ^**c**^			
Keratinizing	888	12.95 ± 3.36	0.0044^*^
Non-keratinzing	552	15.61 ± 3.04	
**Tumor size**			
≤2 cm	23	12.83 ± 3.27	0.0011^*^
>2 cm	7	17.57 ± 1.13	
**Clinical stage**			
I	9	9.78 ± 2.11	<0.0001^*^
II	4	14.25 ± 2.87	
III	5	15.60 ± 2.51	
IV	12	16.25 ± 2.01	
**Lymph node metastasis**			
Negative	24	13.29 ± 3.62	0.048^*^
Positive	6	16.50 ± 1.64	

^*a*^STK33 IHC score showed as mean ± standard deviation.

^*b*^Man-Whitney *U* test for two groups or one-way ANOVA for > two groups.

^*c*^The number was fields of observation by microscope.

*Statistically significant, *P* < 0.05.

### Immunohistochemistry (IHC) and haematoxylin and eosin (HE) staining

Tumor tissues and patient-matched normal hypopharyngeal tissues were fixed in 10% neutral-buffered formalin and embedded in paraffin blocks. 4-μm serial sections were cut, then, IHC and HE staining were carried out. Briefly, tissue sections were performed with citrate buffer (PH = 6.0) and 1.5% hydrogen peroxide in methanol. Slides were incubated with anti-STK33 (1:100, Santa Cruz, CA, USA) at 4°C overnight, then, with second antibody (ZSGB, Beijing, China) at room temperature for 1 h. 3,3’-diaminobenzidine (DAB) was used for visualization of the immunoreaction. As for the HE staining, the sections were stained with hematoxylin and eosin respectively. The process of dehydration, transparence and mounting was similar to that of IHC.

A semiquantitative scoring system named “H-score approach” was used to evaluate the expression level of STK33 [15]. The proportion of epithelial cells staining positively was termed category A and was assigned scores from 1 to 6 (A = 1 (0-4%); 2 (5-19%); 3 (20-39%); 4 (40-59%); 5 (60-79%); 6 (80-100%). Intensity of staining was termed category B and was scored as 0 (negative); 1 (weak); 2 (moderate); 3 (strong). For the H-score assessment, all fields were observed at × 400 magnification [16]. A final score was calculated by multiplying A by B (minimum 0, maximum 18). Meanwhile, the average score of each case was obtained.

### Cell culture

The human HSCC cell line, Fadu cells, was purchased from Biosis (Shanghai, China). Fadu cells were cultured in Dulbecco’s modified Eagle’s medium supplemented with 10% fetal bovine serum at 37°C in a humid atmosphere containing 5% CO_2_. Cells were treated with 1 to 10 μM PD98059 (an inhibitor of ERK1/2, Sigma, MO, USA).

### Construction of STK33-RNAi lentiviral vector and transfection into Fadu cells

A STK33-RNA interference (STK33-RNAi) lentiviral vector (GV115-GFP-STK33 shRNA) was constructed by GeneChem Co, Ltd (Shanghai, China) and a GFP-lentiviral vector (GV115-GFP) was used as a negative control (Mock RNAi). In brief, after identification of the target sequence of STK33-RNAi (NM_030906, CTCAAGAACCTCAAATGTA), double-stranded oligonucleotides encoding human STK33-vshRNA were annealed and inserted into the small hairpin RNA (shRNA) expression vector GV115-GFP. Next, the lentiviral titers of STK33-RNAi and control mock RNAi were identified. In order to detect the efficiency of transfection in Fadu cells, 2 × 10^4^ Fadu cells were seeded into 15 mm plate specially used for laser scanning confocal microscope and incubated for 24 h. GV115-GFP-STK33-shRNA and GV115-GFP, which were diluted with 270 μl 10% FBS and 30 μl polybrene (50 μg/ml, Sigma, MO, USA), pretreated Fadu cells for 12 h. Subsequently, the solution was replaced with 10% FBS-DMEM. Transfected Fadu cells were observed with laser scanning confocal microscopy everyday.

### Assessment for cell viability

The 3-(4, 5-Dimethylthiazol-2-yl)-2, 5-Diphenyltetrazolium Bromide (MTT) assay was used to evaluate the cell viability. In brief, 3 × 10^4^/ml (100 μl per well) Fadu cells were seeded in 96-well culture plate and incubated for 24 h, then, replaced with fresh medium containing different concentrations of PD98059 (0, 1, 5, and 10 μM) for another 24, 48 and 72 h incubation, respectively. With respect to the effect of STK33-RNAi related groups, after Fadu cells were transfected with mock RNAi and 1 × 10^7^ TU/ml STK33-RNAi for 48 h, cells were subjected to 0 and 5 μM PD98059 for another 48 h. Then, 20 μl MTT (5 mg/ml) was added into each well and incubated at 37°C for additional 4 h. Subsequently, the supernatant was removed, 100 μl DMSO was added into each well and shaken for another 20 min until the crystals were dissolved. The optical density (OD) values were measured at 570 nm using an ELISA reader (Multiskan MK3, Finland). Cell viability was calculated according to the following formula:

$$ Cell\  relative\kern0.3em  viability\ \left(\%\right) = O{D}_{experiment}/O{D}_{control}\times 100\%\left(O{D}_{\mathrm{blank}}\mathrm{is}\ \mathrm{adjusted}\ \mathrm{t}\mathrm{o}\ \mathrm{zero}\right) $$

### Morphological examination by Ho.33342 (HO) and Propidium Iodide (PI) double fluorescent staining

2 × 10^4^ Fadu cells/ml were seeded in dishes (20 mm) for 24 h. Cells were transfected with mock RNAi and 1 × 10^7^ TU/ml STK33-RNAi for 48 h followed by the administration of 0 and 5 μM PD98059 for another 48 h. Then, cells were washed twice by PBS, fixed with 95% alcohol for 10 min, and stained by Ho (10 μg/ml) and PI (50 μg/ml) at 37°C for 30 min. Laser scanning confocal microscopy was used to observe the morphological changes of Fadu cells with green light (488 nm) for blue nucleus and red light (532 nm) for red cells respectively, later, the two pictures were integrated together as one. The number of dead cells within every 200 cells were counted [17].

### Colony formation assay

500 cells/per pore were seeded into 6-well plate and cultured for 24 h. Next, cells were transfected with mock RNAi and 1 × 10^7^ TU/ml STK33-RNAi for 48 h and cells were treated with 0 and 5 μM PD98059 every 3 d till 10 d. Then, cells were washed twice with PBS, fixed with 95% alcohol for 10 min and stained with crystal violet for 10 min, subsequently, washed with water till the plates were clear. Finally, the number of colonies where the number of cells exceeded 50 was counted [18].

### Assessment of cell migration and invasion

A Costar Transwell chamber (Corning, NY, USA) was employed to assess cell migration. As for cell invasion assay, the membrane was covered with matrigel (BD, MA, USA). Firstly, Fadu cells were transfected with mock RNAi and 1 × 10^7^ TU/ml STK33-RNAi for 48 h. Then, 100 μl (1 × 10^4^/ml) cell suspension with serum-free DMEM, which simultaneously contained PD98059 at final concentrations of 0 and 5 μM, was individually added into the upper chamber and 500 μl complete medium was consistently placed into the lower chamber. These cells were incubated at 37°C for 48 h, washed with PBS and fixed by methanol. Then, cells remaining on the upper surface of the filter membrane were completely removed, while, the cells still on the opposite surface of the filter membrane were stained with crystal violet for 10 min. The migratory cells were then observed and counted in six random fields (Magnification 100×).

### mRNA extraction and real-time PCR analysis

Fadu cells (1.5 × 10^5^ cells/ml, 1 ml) were cultured in 6-well culture plates for 24 h. Then, cells were transfected with mock RNAi and 1 × 10^7^ TU/ml STK33-RNAi lentiviral vector for 48 h and cells were treated with 0 and 5 μM PD98059 for another 48 h. These cells were collected for extracting the total RNA with Trizol (Invitrogen, USA). Then, mRNA was reverse transcribed into cDNA (Fermentas, Ontario, Canada) and SYBR Green real-time PCR (Takara, Dalian, China) assay was employed. PCR primers (Sangon Biotech, Shanghai, China) for the genes were listed in Table 2. The conditions were as follows: pre-degeneration at 95°C for 2 min, degeneration at 95°C for 15 sec, renaturation at different temperatures (Table 2), elongation at 72°C for 40 sec, total 40 cycles. The data were analyzed using the Realplex2 Real-Time PCR System (Eppendorf, Hamburg, Germany). The real-time PCR experiments with every gene were carried out in triplicate, and Mean ± SEM was used for the determination of mRNA levels. By use of the comparative Ct method with GAPDH as the reference gene, relative quantification of the mRNA levels was performed with the formula 2^-△△Ct^.

**Table 2:** **Primer, amplicon size and annealing temperature of each gene for qRT-PCR**

Gene	Forward 5′to3′	Reverse 5′to3′	Product length/bp	Tm/°C	Genbank NCBI reference sequence
STK33	CCCCGACTGTTCATCTGCTTCT	TGCCCACTTCGTTTCTGTTTCC	391	54	Nm_030906.2
ERK1/2	TCCAAGGGCTACACCAAGTC	CTCGTCACTCGGGTCGTAAT	363	60	Nm_002745.4
Caspase-3	CAGAACTGGACTGTGGCATTG	GCTTGTCGGCATACTGTTTCA	192	54	Nm_004346.3
Bcl-2	GTGGCCTTCTTTGAGTTCGG	TCCACAGGGCGATGTTGTC	88	55	Nm_000633.2
Ki-67	TGTCACATCGCTATTTCAAATTCAG	GGAGGGCTTGCAGAGCATTTATC	170	54	Nm_001145966.1
E-Cadherin	GGGGTCTGTCATGGAAGGTGCT	GTAAGCGATGGCGGCATTGTAG	103	57	Nm_004360.3
Vimentin	AGGCAGAAGAATGGTACAAATCC	TTTAAGGGCATCCACTTCACAG	146	55	Nm_003380.3
Nm-23-H1	CTGGGACCATCCGTGGAGACT	TCCTCAGGGTGAAACCACAAGC	112	55	Nm_000269.2
GAPDH	AGGTCGGTGTGAACGGATTTG	TGTAGACCATGTAGTTGAGGTCA	130	55	Nm_001289746.1

### Protein extraction and western blot analysis

Fadu cells (1.5 × 10^5^ cells/ml, 1 ml) were cultured in 6-well culture plates for 24 h. Subsequently, cells were transfected with mock RNAi and 1× 10^7^ TU/ml STK33-RNAi lentiviral vector for 48 h and cells were treated with 0 and 5 μM PD98059 for another 48 h. These cells were used to extract the total protein with lysis buffer (PMSF: RIPA = 1:100) and the concentration of protein was measured with the BCA protein assay kit (Beyotime, Shanghai, China). 35 μg protein was separated by 6% (Ki-67), 10% (E-Cadherin), 12.5% (Vimentin, STK33, β-Actin, ERK1/2) and 15% (Caspase-3, Bcl-2, Nm-23-H1) SDS-PAGE gels. After electrophoresis, transmenbrane and blockage, the PVDF membranes (Millipore, USA) were incubated with primary antibodies overnight at 4°C (anti-STK33: 1:400, anti-Caspase-3: 1:400, anti-Bcl-2: 1:400 (Santa Cruz, CA, USA), anti-ERK1/2: 1:400, (Abcam, Cambridge, USA), anti-E-Cadherin: 1:300, anti-Vimentin: 1:10000, anti-Nm-23-H1: 1:2000, (Epitomics, Calfornia, USA), anti-Ki-67: 1:500, and anti-β-Actin: 1:2000 (Biosynthesis, Beijing, China). Then, the membranes were incubated with the secondary antibody (1:2000) at room temperature for 1 h. Finally, the immunoblots were developed using ECL reagents (Santa Cruz, CA, USA) and detected after exposure to X-ray film. Densitometer readings were carried out to quantitate the immunoreactive bands. The ratios of related-genes to β-actin were then determined.

### Statistical analysis

The STK33 IHC scores of each subgroup of clinicopathological parameters in Table 1 were shown as mean ± standard deviation (SD). The Man-Whitney *U* test was applied for comparisons between two groups, while, one-way analysis of variance (ANOVA) was used to compare more than two groups. The other data were presented as mean ± standard error of mean (SEM) of separate experiments (n ≥ 3) and statistically analyzed by one-way ANOVA. Statistical calculations were performed using SPSS software package for Windows (version 13.0; SPSS, Chicago, IL). *P* value of less than 0.05 was considered significant.

## Results

### STK33 expression increased in human HSCC specimens and activated in Fadu cells

**Figure 1:**
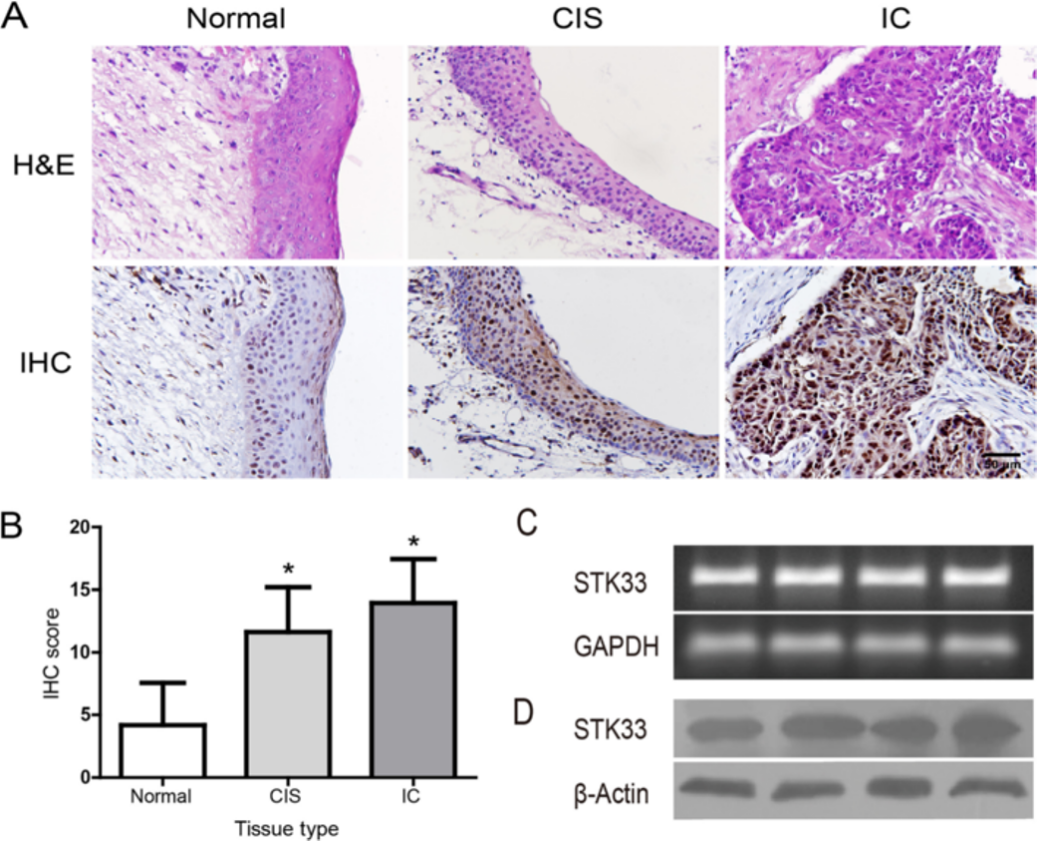
**Expression of STK33 in human HSCC tissue and Fadu cells. (A)** Representative imagines of STK33 IHC staining and matched H&E staining in hypopharyngeal normal and tumor tissues. a) Low STK33 expression in normal tissue; b) Moderate STK33 expression in CIS; and c) High STK33 expression in IC (Magnification × 400, Scale bars, 50 μm) **(B)** IHC score of STK33 expression in samples shown in **A**. Data were expressed as the mean ± SD. * P < 0.05. **(C, D)** The STK33 mRNA and protein were expressed abundantly in Fadu cells as determined by qRT-PCR and western blot.

The expression of STK33 in human normal and hypopharynx tumor tissue was examined by IHC. As shown in Figure 1A, B STK33 protein was localized in nucleus and, partly, in cytoplasm in all specimens. With respect to the staining intensity, normal tissue, cancer in situ (CIS), and invasive cancer (IC) displayed weak, moderate and strong immunoreactivity for STK33 protein, respectively. STK33 IHC score was significantly decreased in normal tissue (4.17 ± 3.38) compared with that in CIS (11.63 ± 3.56, *P* < 0.05) or IC (13.97 ± 3.47, *P* < 0.05). In contrast, there was statistical difference in STK33 IHC score between CIS and IC (*P* < 0.05).

Moreover, the basic status of STK33 in Fadu cells was initially examined at both mRNA and protein levels. As shown in Figure 1C, D STK33 mRNA expression in Fadu cells was apparent from the RT-PCR data and, correspondingly, similar situation for STK33 protein expression was found by western blot.

### Correlation of STK33 expression with clinicopathological characteristics

**Figure 2:**
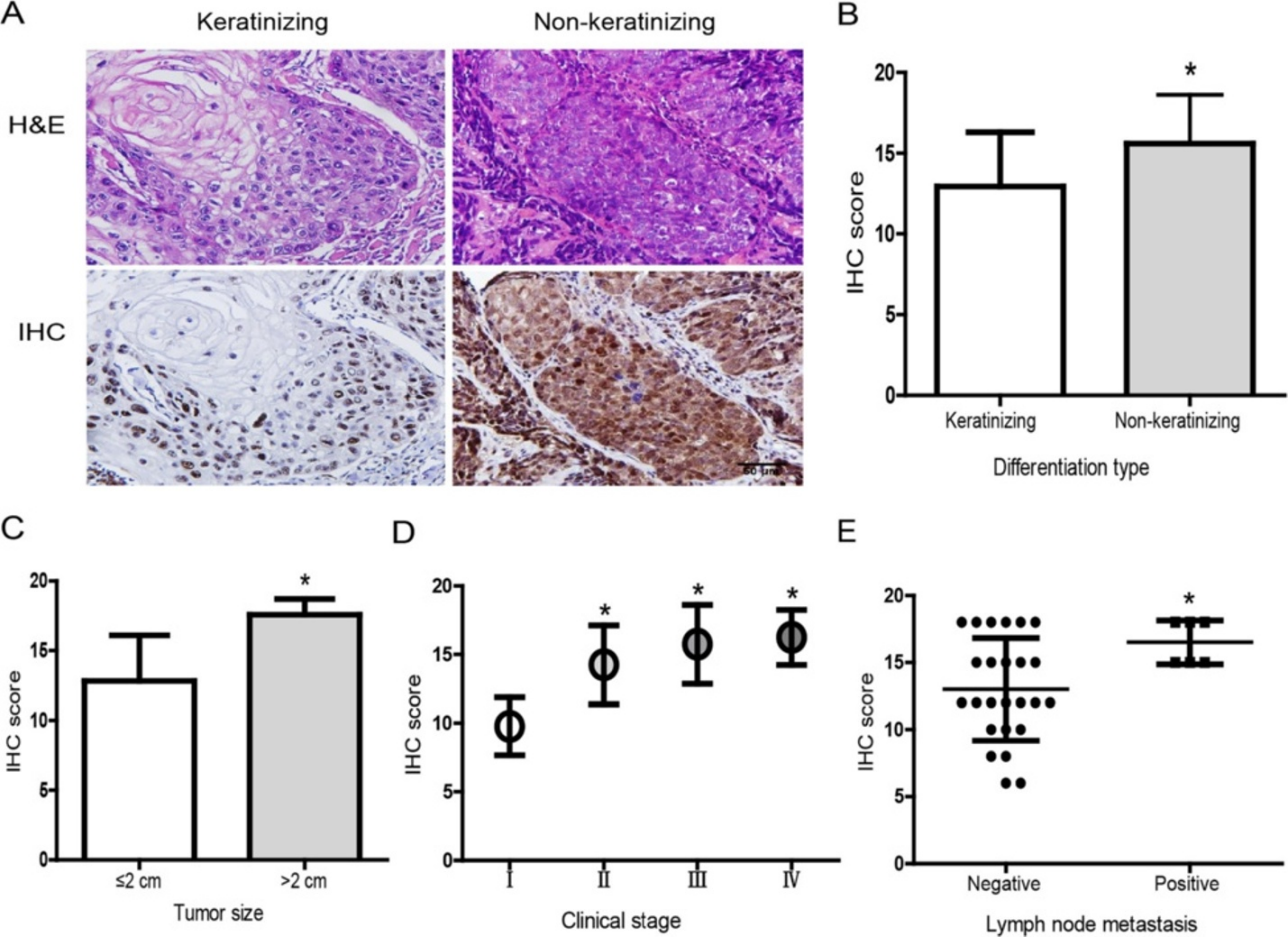
**Correlation of STK33 expression with clinicopathological parameters. (A)** Representative images of STK33 IHC staining in different differentiation types and corresponding H&E staining (Magnification × 400, Scale bars, 50 μm). **(B)** STK33 IHC score was significantly higher in non-keratinizing type than that in keratinizing type shown in **A**. Moreover, STK33 IHC score was significantly correlated with tumor size **(C)** clinical stages **(D)** lymph node metastasis **(E)**. Data were expressed as the mean ± SD. * P < 0.05.

The clinicopathological characteristics of the patients with HSCC and analyses of STK33 immunostaining were summarized in Table 1 (Figure 2). Histopathologically, the tumors are traditionally graded into keratinizing and non-keratinizing SCC. Keratinizing type is characterized by large tumor island with variable “pearl” formation pushing margins. Non-keratinizing SCC is characterized by scattered small irregular cords or single tumor cells, with poorly defined infiltrating margins [17]. In the present study, IHC staining showed that the intensity of STK33 expression was much stronger in non-keratinizing type than keratinizing type of HSCC, with a statistically significant difference in IHC score between the two different types (*P* < 0.05). In addition, STK33 IHC scores were markedly increased in HSCC specimens in size >2 cm, compared with those ≤ 2 cm (*P* < 0.05). Also, IHC score of STK33 was significantly higher in patients at advanced HSCC stages (stageII, III and IV) than that in stageI (*P* < 0.05). Similarly, IHC score was significantly increased with metastasis to the lymph nodes compared with non-metastasis (*P* < 0.05). There were no other significant correlations between STK33 expression with patient age or with sex.

### Effects of STK33-RNAi and/or PD98059 on cell viability, apoptosis and colony formation in Fadu cells

**Figure 3:**
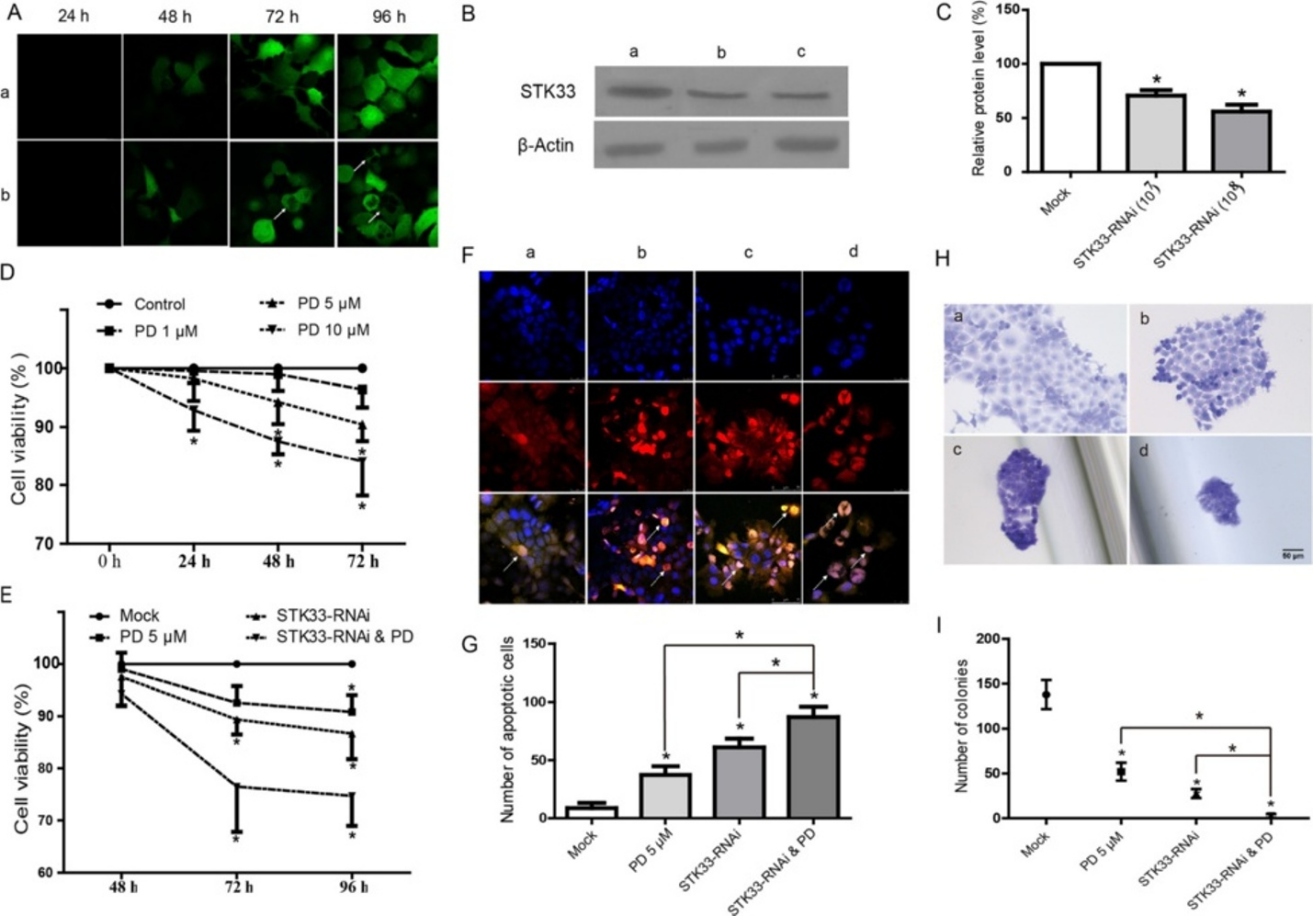
**Effects of STK33-RNAi and PD98059 on Fadu cells and in vitro cell function test. (A)** Cells with GFP signal were considered successfully transfected. Increase of the green fluorescence in Fadu cells transfected with both STK33-RNAi and scrambled RNAi was correlated with the post-transfection time, however, apoptosis (↑) was induced only in the cells subjected to STK33-RNAi (Magnification × 400). a) The scrambled RNAi, 1 × 10^7^ TU/ml, b) The STK33-RNAi, 1 × 10^7^ TU/ml. **(B)** Transfection of STK33-RNAi into the cells led to a significant decrease in STK33 protein expression compared with that in the mock-transfected cells. a) The scrambled RNAi, 1 × 10^7^ TU/ml, b) STK33-RNAi, 1 × 10^7^ TU/ml, c) STK33-RNAi, 1 × 10^8^ TU/ml. **(C)** Data represent the mean ± SEM from three western blot analyses * P < 0.05.**(D, E)** MTT assay showed that the decrease in cell viability with PD98059 appeared in a concentration- and time-manner and the decrease in cell viability with STK33-RNAi was correlated with the post-transfection time. The effect exerted by STK33-RNAi was enhanced by PD98059. In addition, induction of apoptosis (↑) and reduction of colony size in Fadu cells by STK33-RNAi and PD98059 were assessed by Ho.33342 and PI staining (**F**, Magnification × 200), and colony formation (**H**, Magnification × 100, Scale bars, 50 μm), respectively. a) Control, b) 5 μM PD98059, c) STK33-RNAi, d) STK33-RNAi and 5 μM PD98059. Quantitative analyses of the number of apoptotic cells **(G)** and colonies **(I)** in response to the different stimuli were shown. Data represented the mean ± SEM; n = 3, * P < 0.05.

As shown in Figure 3A-C, green fluorescence in Fadu cells was increased in a time-manner after the transfection. Meanwhile, cells subjected to STK33-RNAi showed the reduction in cell volume, chromatin condensation, and/or presence of apoptotic bodies, which were the typical morphological features of apoptosis. Whereas, cells transfected with scrambled RNAi showed regularly polygonal shape, the normal morphology of Fadu cells. These results indicated that STK33-RNAi lentiviral vector was not only successfully transfected into Fadu cells, but exerted effective function on the cells as well. Western blot assay further showed the expression of STK33 in cells transfected with STK33-RNAi was significantly decreased than that in cells transfected with scrambled RNAi (*P* < 0.05), which suggested that STK33-RNAi effectively suppressed STK33 expression in Fadu cells.

As shown in Figure 3D, E the cell viability significantly decreased with increase in the concentrations of PD98059 and the post-treatment time (*P* < 0.05) by MTT assay. The obtained results revealed that exposure of Fadu cells to 5 μM PD98059 for 48 h exhibited the least effective response. Furthermore, the exposure of Fadu cells to STK33-RNAi and/or PD98059 resulted in a marked reduction of cell viability (*P* < 0.05), and of note, the effect by STK33-RNAi was significantly potentiated by addition of PD98059 (*P* < 0.05).

The effects of STK33-RNAi and PD98059 on morphological changes of Fadu cells were examined with the dual staining of HO and PI. As illustrated in Figure 3F, G the typical characteristics of apoptosis were presented, including cell shrinkage, chromatin condensation, and apoptotic bodies. Quantitative analyses of the number of apoptotic cells revealed that both STK33-RNAi and 5 μM PD98059 led to a significant increase in apoptotic cells in comparison to that in control group (*P* < 0.05) and, meanwhile, the number was markedly higher in group with STK33-RNAi in combination with 5 μM PD98059 than that in group exposure to anyone alone (*P* < 0.05), indicating that STK33-RNAi and PD98059 cooperatively induced apoptosis in Fadu cells.

As depicted in Figure 3H, I the colony size of cells subjected to any intervention was smaller than that of control cells. In addition, quantitative analyses revealed that the numbers of the formed colonies were markedly reduced by STK33-RNAi, PD98059, and a combination of both, compared with that by mock-RNAi. Of these interventions, the combination of STK33-RNAi with PD98059 led to form minimum number of clones, thereby displaying a synergistic effect of STK33-RANi and PD98059 on colony formation of Fadu cells.

### Effects of STK33-RNAi and/or PD98059 on mRNA and protein expressions of Caspase-3, Bcl-2 and Ki-67 in Fadu cells

**Figure 4:**
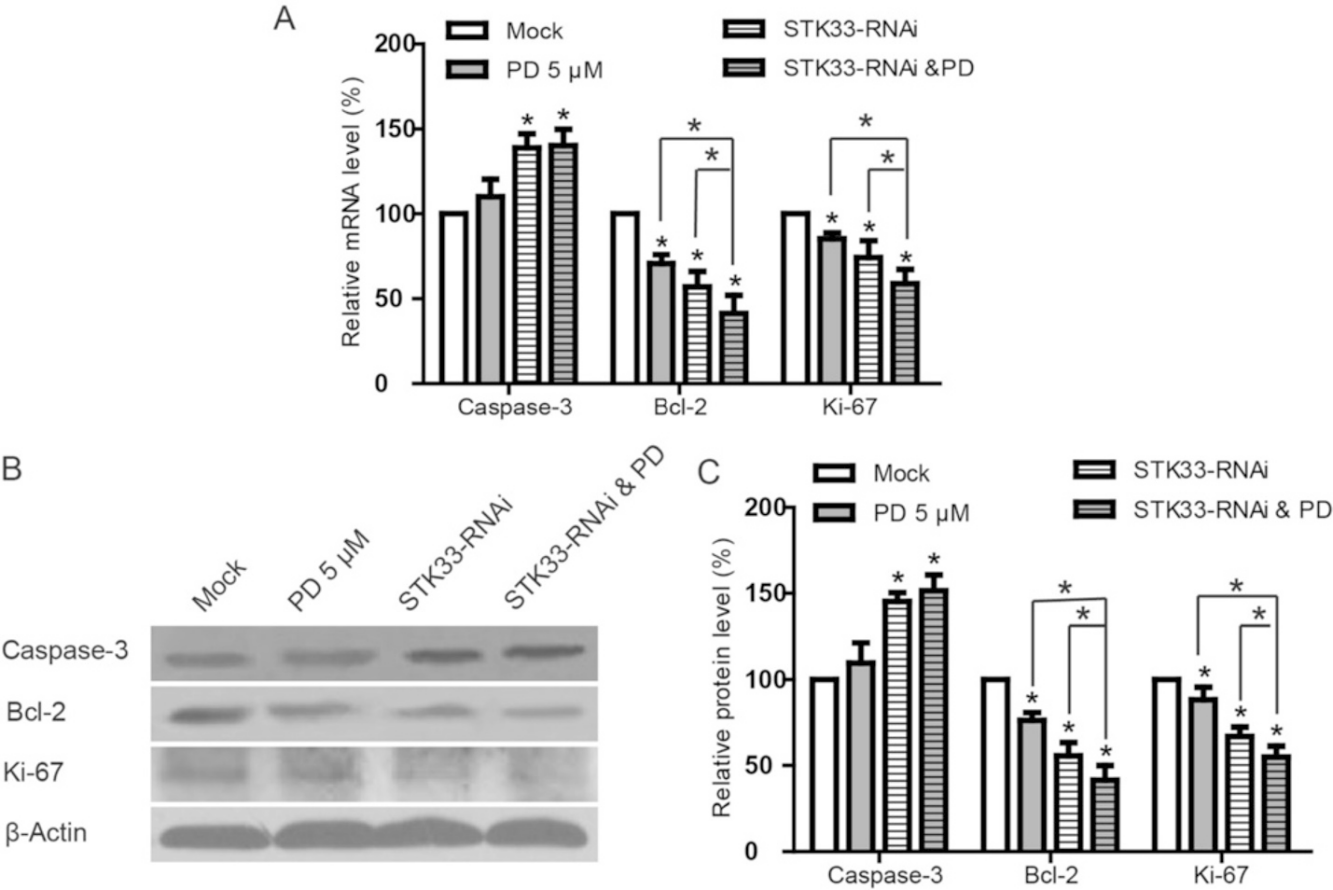
**Effects of STK33-RNAi and/or PD98059 on mRNA and protein expressions of genes relevant to apoptosis and proliferation of Fadu cells.** Real-time PCR **(A)** and western blot **(B, C)** showed STK33-RNAi significantly increased the expression of Caspase-3 in Fadu cells at 96 h post-transfection. Meanwhile, STK33-RNAi markedly decreased the expressions of Bcl-2 and Ki-67 in Fadu cells, and the effects were potentiated by PD98059. Results were shown as the means ± SEM, n = 3. *P < 0.05.

As both STK33-RNAi and PD98059 can induce apoptosis in Fadu cells, we further investigated the possible mechanisms underlying this action via examination of activities of certain typical genes relevant to apoptosis or proliferation with quantitative RT-PCR and western blot. STK33-RNAi markedly increased the mRNA and protein expressions of Caspase-3 in Fadu cells at 96 h post-transfection and 5 μM PD98059 led to a trend towards increase in Caspase-3 expression of but not reaching significance (*P* > 0.05) compared with control. On the other hand, a significant reduction of Bcl-2 and Ki-67 expressions in Fadu cells after exposure to STK33-RNAi or 5 μM PD98059 were detected, compared with those in control groups (*P* < 0.05). Furthermore, Bcl-2 and Ki-67 expressions in Fadu cells subjected to STK33-RNAi plus 5 μM PD98059 were lower than those in the cells to treatment alone (*P* < 0.05). These data indicated that STK33-RNAi and PD98059 exerted synergistic effects on the expressions of these aforementioned genes (Figure 4).

### Effects of STK33-RNAi and/or PD98059 on the suppression of migration and invasion in Fadu cells, and the expressions of relevant genes

**Figure 5:**
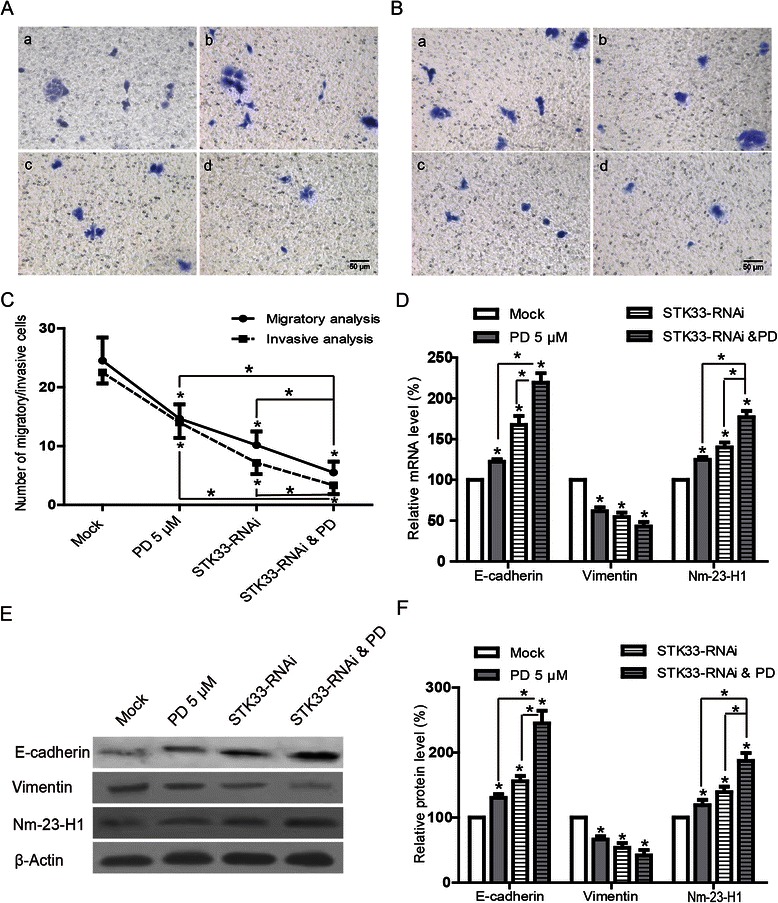
**Effects of STK33-RNAi and PD98059 on migratory and invasive abilities of Fadu cells and relevant genes.** Representative images of cystal violet-stained migratory **(A)** and invasive **(B)** cells after exposure to the scrambled RNAi and STK33-RNAi, respectively (Magnification × 100, Scale bars, 50 μm). a) Mock, b) 5 μM PD98059, c) STK33-RNAi, d) STK33-RNAi plus 5 μM PD98059. **(C)** Numbers of migratory and invasive cells in response to different interventions. STK33-RNAi significantly increased the mRNA **(D)** and protein **(E, F)** expressions of E-Cadherin and Nm-23-H1, while, PD98059 enhanced the effects and STK33-RNAi obviously decreased the Vimentin expressions. Results were shown as means ± SEM, n = 3. *P < 0.05.

To explore the effects of STK33 on migratory and invasive activities of Fadu cells, *in vitro* invasion assessment was performed using the Transwell assay. As shown in Figure 5A-C, the number of migratory cells were significantly reduced by both STK33-RNAi and PD98059 compared with that with scrambled RNAi (*P* < 0.05) and, moreover, the effect by STK33-RNAi was significantly potentiated by addition of PD98059 (*P* < 0.05). With respect to the effect of STK33-RNAi on invasion, similar results were achieved by this assay. These data implied that STK33-RNAi compromised the migratory and invasive capacity of Fadu cells.

As shown in Figure 5D-F, treatment of Fadu cells with STK33-RNAi or 5 μM PD98059 significantly promoted the E-Cadherin expression (*P* < 0.05) and markedly inhibited the Vimentin expression (*P* < 0.05) compared with that in control. Simultaneously, STK33-RNAi plus 5 μM PD98059 remarkably induced the E-Cadherin expression in Fadu cells than that in group subjected to single intervention (*P* < 0.05). As for the expression of Nm-23-H1, either STK33-RNAi or 5 μM PD98059 similarly resulted in an obvious elevation at mRNA and protein levels in Fadu cells compared with that in control (*P* < 0.05). Moreover, Nm-23-H1 expression was markedly higher in STK33-RNAi and 5 μM PD98059 group than that in any one group (*P* < 0.05).

### Effect of STK33-RNAi and/or PD98059 on STK33 and ERK1/2 expressions in Fadu cells

**Figure 6:**
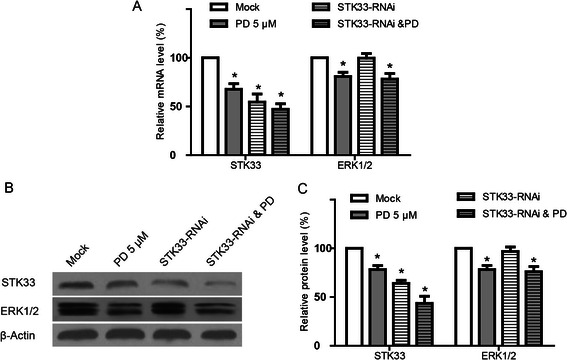
**Alterations in mRNA and protein expressions of STK33 and ERK1/2 in Fadu cells in response to STK33-RNAi and/or PD98059.** Either real-time PCR **(A)** or western blots **(B,C)** showed that PD98059 markedly decreased both STK33 and ERK1/2 levels in Fadu cells, but, STK33-RNAi only significantly diminished STK33 expressions. Data showed the mean ± SEM, n = 3, *P < 0.05.

To further determine the relationship between STK33 and ERK1/2 signaling pathway, the mRNA and protein expressions of STK33 and ERK1/2 in Fadu cells in response to STK33-RNAi and/or PD98059 were examined by quantitative RT-PCR and western blot in Fadu cells. As shown in Figure 6, PD98059 significantly inhibited ERK1/2 expressions at both mRNA and protein levels in Fadu cells compared with those in the scrambled RNAi-tansfected cells and, also, PD98059 markedly reduced STK33 expression (*P* < 0.05). On the other hand, STK33-RNAi significantly decreased the mRNA and protein expressions of STK33 (*P* < 0.05) and, STK33-RNAi, however, had no impact on ERK1/2 expression (*P* > 0.05).

## Discussion

Previous studies on human somatic tumors have shown that STK33 acts as a novel tumor gene [4,7,8]. However, these few data published relevant to STK33 expression in different types of tumor are conflicting and the possible role of STK33 on human HSCC has not been investigated to date. In the present study, strong STK33 immunostaining was noted in human HSCC, but, weak staining was presented in corresponding normal tissues. This indicates that STK33 over-expression may play a crucial role in the pathogenesis of HSCC, which is consistent with the findings in other types of tumor [4,7,19]. Meanwhile, we found that STK33 was abundantly expressed at both mRNA and protein levels, confirming the activation of STK33 in Fadu cells. Keratinizing HSCC is well differentiated and associated with a better prognosis, whereas, non-keratinizing HSCC is poorly differentiated and associated with a more aggressive course [13]. In the present work, the expression of STK33 was markedly elevated in non-keratinizing type than that in keratinizing type. Therefore, the significance of the greater expression of STK33 in non-keratinizing HSCC than in keratinizing type is that STK33 might be related to the dedifferentiation in HSCC. Moreover, statistical analyses showed that there existed significant associations between STK33 expression in HSCC with tumor size, clinical stage, and lymph node metastasis. These results indicate that aberrant expression of STK33 may promote the initiation and progression of HSCC. Our findings highly support an oncogenic role of STK33 in human HSCC, which sheds a new light on the mechanisms underlying the occurrence of HSCC.

To examine the effect of STK33 *per se*, the STK33 RNAi was generated and introduced into Fadu cell. We found that the cells transfected with STK33 RNAi not only exhibited a marked reduction in STK33 protein expression, but also appeared the morphological change compared with those in the mock RNAi-transfected cells, indicating that the generated STK33 RNAi was effective. Next, we concentrated on exploring whether STK33 knockdown alone or together with PD98059, had the potential to alter the properties of Fadu cells and, if so, the possible mechanism underlying such an action.

In this work, we demonstrated that either STK33-RNAi or PD98059 remarkably reduced cell viability of Fadu cells, implying that STK33-RNAi was able to inhibit the growth of Fadu cells *in vitro*. Meanwhile, the exposure of Fadu cells to STK33-RNAi led to cell destruction with specific morphologic features, which were in accordance with typical morphological characteristics of cells undergone apoptosis. This indicates that STK33-RNAi exerts its cytotoxity on Fadu cells is mainly attributable to the induction of apoptosis. Not only that, this cytotoxic effect was potentiated by the administration of PD98059 at the minimum dose, exhibiting a synergistic effect of the two insults. Moreover, the ability of colony formation is necessary for a tumor cell growing to a micro- lesion and furthermore developing into macro-lesion [20]. We found STK33-RNAi caused a marked reduction in colony number of Fadu cells, which means STK33-RNAi may suppress the coloning proliferation of Fadu cells, while, PD98059 may potentiate the effect. These data reveal that STK33 is an important factor and that a cross-talk network exists between STK33 and ERK1/2 in the regulation of survival and proliferation in Fadu cells.

It has been well known that cytochrome c is released from mitochondria to cytoplasm, which in turn activates a series of other apoptosomes and, particularly, the activation of Caspase-3 responsible for the execution of the apoptotic program in response to apoptotic signals [21]. In the present study, the activities of Caspase-3 were remarkably up-regulated by STK33-RNAi, suggesting that STK33 knockdown -induced apoptosis in Fadu cells is depend on mitochondrial pathway. It is well known that proteins of the Bcl-2 family, which generally either repress apoptosis or promote apoptosis, play a key role in controlling the activation of caspases [22]. In this work, a decrease in expression of Bcl-2 were observed after exposure to STK33-RNAi, indicating that STK33 knockdown triggers apoptosis by down-regulating the activities of anti-apoptotic Bcl-2, thereby revealing another mechanism underlying the action of STK33 knockdown on Fadu cells. As Ki67 universally expresses among proliferating cells and is absent in quiescent cells, it is commonly used as a marker for the evaluation of cell proliferation [23]. We found that STK33-RNAi significantly suppressed Ki-67 expressions at mRNA and protein levels, further revealing the inhibitory trait of STK33 knockdown on Fadu cells. In addition, PD98059 acts cooperatively with STK33-RNAi to affect the expressions of these genes, providing additional proof for a cross-talk interaction between STK33 and ERK1/2 in Fadu cells.

Tumor cell invasion and metastasis are complex processes that involve multiple steps [19]. In the present investigation, STK33 knockdown altered the migratory and invasive phenotype of the cells. Similarly, such effects by STK33-RNAi were enhanced with PD98059. Epithelial-mesenchymal transition (EMT) is a process by which epithelial cells lose their polarity and are converted to a mesenchymal phenotype, which represents an important pathway to mediate invasion and metastasis of tumors [24]. A defining feature of EMT is a reduction in E-Cadherin level and a concomitant induction of higher expression of Vimentin [25]. It has been documented that ERK1/2 pathway is involved in EGF-induced EMT and PD98059 diminished the EGF-induced EMT in SBOT cells [26,27]. As for the relationship between STK33 and EMT, it was reported that STK33 exhibited autophosphorylation and phosphorylated the intermediate filament protein Vimentin directly [28]. Our data revealed STK33-RNAi not only blocked Vimentin expression but also simultaneously elevated E-Cadherin expression, implying STK33-RNAi suppresses EMT through both of the two aspects. Furthermore, the synergistic effects of STK33-RNAi and PD98059 on the expressions of Vimentin and E-Cadherin exist, suggesting STK33 may trigger EMT involved in EKR1/2 pathway. Lastly, we demonstrated STK33-RNAi significantly increased the Nm-23-H1 expression and the effect was enhanced by PD98059. This suggests that STK33-knockdown inhibits the invasion and metastasis of Fadu cells via triggering the activity of Nm-23-H1 gene, which is well known to possess anti-metastasis trait in tumors [29]. These data indicate that STK33 may promote metastasis in HSCC and increase migration and invasion in Fadu cells via inducing EMT by activating Vimentin and suppressing E-Cadherin, and reducing Nm-23-H1 genes, while, EKR1/2 pathway is involved in the process.

In view of the aforementioned findings that STK33-RNAi and PD98059 act in close collaboration each other on Fadu cells, we tried to seek out the direct evidence in relation to STK33 and ERK1/2 pathway by the examination of STK33 and ERK1/2 expressions. In the present study, we found that STK33-RNAi only markedly suppressed STK33 expression but failed to impact ERK1/2 expression; however, PD98059 significantly reduced both ERK1/2 and STK33 expressions at mRNA and protein levels in Fadu cells. These findings suggest that ERK1/2 might directly mediate STK33 activity, implying that STK33 is possibly a downstream of ERK1/2, which is consistent with the previous findings [4]. However, it has been reported that reduction of STK33 has no effect on the expression of ERK1/2 [9], indicating that the exact relationship between STK33 activity and ERK1/2 pathway needs to be deciphered further.

## Conclusions

In conclusion, we identify that STK33 is a potential oncogene and may be useful as a diagnostic marker for HSCC. The findings from this work highlight the functional role of STK33 that inhibits apoptosis, enhances proliferation, promotes metastasis and invasion, as well as triggers EMT, which is possibly related to ERK1/2 pathway. Thus our study illuminates a novel axis that STK33 may induce tumorigenesis of human HSCC, and, perhaps, serve as a valuable molecular target for treatment of HSCC. Further study should be focused on the identification of a direct substrate for STK33 so as to determine the precise role of STK33 gene in the initiation and progression of cancer cells.

## Abbreviations

STK33: Serine/threonine kinase 33
HSCC: Hypopharyngeal squamous cell carcinoma
CAMK: Calcium/calmodulin dependent kinase
MAPK: Mitogen-activated protein kinase
ERK1/2: Extracellular signal-regulated protein kinases 1 and 2
EMT: Epithelial mesenchymal transition
IHC: Immunohistochemistry
HE: Haematoxylin and eosin
STK33-RNAi: STK33-RNA interference
shRNA: Small hairpin RNA
MTT: 3-(4, 5-Dimethylthiazol-2-yl)-2, 5-Diphenyltetrazolium Bromide
OD: Optical density
HO: Ho.33342
PI: Propidium iodide
SD: Standard deviation
SEM: Standard error of mean
ANOVA: Analysis of variance
CIS: Cancer in situ
IC: Invasive cancer
